# Comment on Golčić et al. Evaluation of Systemic Treatment Options for Gastrointestinal Stromal Tumours. *Cancers* 2023, *15*, 4081

**DOI:** 10.3390/cancers15235618

**Published:** 2023-11-28

**Authors:** Anni Chen, Xinhua Zhang

**Affiliations:** Department of Gastrointestinal Surgery, The First Affiliated Hospital of Sun Yat-sen University, Guangzhou 510080, China; chenann6@mail2.sysu.edu.cn

We carefully read the article written by Golčić et al. “Evaluation of Systemic Treatment Options for Gastrointestinal Stromal Tumours” [1]. The authors included the dose escalation of imatinib as an option for the second-line treatment of metastatic or unresectable GISTs. Given that the definition of second-line treatment provided by the authors may be problematic, we have once again referred to the ESMO guidelines (2021) cited in the article [2]. The guidelines explicitly state that “In the case of confirmed progression or rare intolerance on imatinib (after attempts to manage side-effects through expert advice, exploiting dose reductions and possibly plasma level assessment), standard second-line treatment is sunitinib”, and there is no mention of the dose escalation of imatinib as part of the second-line treatment. The statement regarding the dose escalation of imatinib treatment in the guidelines is as follows: “In the case of tumour progression on 400 mg, an option may be to increase the imatinib dose to 800 mg daily (if treated with the lower dose), with the exception of insensitive mutations. Dose escalation is particularly useful in the case of a *KIT* exon 9-mutated GIST (if a higher dose was not selected from the beginning) and possibly in the case of fluctuations in drug pharmacokinetics over time”.

In addition, we also compared the NCCN Guidelines and GEIS Guidelines for GISTs [3,4]. In the NCCN guidelines, the table titled ”Systemic Therapy Agents and Regimens for Unresectable, Progressive, or Metastatic Disease” includes sunitinib and ripretinib as preferred second-line treatment options, but not imatinib. The GEIS guidelines also state that sunitinib is the standard second-line treatment option. Imatinib dose escalation can be considered as an option for disease progression after standard-dose imatinib treatment, but it does not fall under the category of second-line treatment, which is consistent with the ESMO guidelines.

Based on the statements provided in the above-mentioned guidelines, we believe that the categorisation of treatment lines for unresectable, progressive, or metastatic GISTs should be based on the switch of agents rather than the adjustment of the dosage of the same agent.

Lastly, I kindly request that the authors consider making the necessary corrections to the error and pay closer attention to accuracy and credibility in future articles. This will ensure that readers receive correct and reliable information.
